# Factors that Influence the Immunological Adjuvant Effect of *Lactobacillus fermentum* PC1 on Specific Immune Responses in Mice to Orally Administered Antigens

**DOI:** 10.3390/vaccines4030024

**Published:** 2016-07-19

**Authors:** Meera Esvaran, Patricia L. Conway

**Affiliations:** Centre for Marine Bio-Innovation, School of Biological, Earth and Environmental Sciences, The University of New South Wales, Sydney, NSW 2052, Australia; M.Esvaran@unsw.edu.au

**Keywords:** *L. fermentum* PC1, adjuvant, OVA, *S*. Typhimurium, oral adjuvant, lactobacillus

## Abstract

This study examined the influences of the dosage of the adjuvant, the nature of the antigen and the host genetics on the capacity of *L. fermentum* PC1 (PC1) to function as an oral adjuvant. BALB/c and DBA/1 mice were vaccinated with either ovalbumin (OVA) or *Salmonella* Typhimurium on days 0 and 14, Mice were also dosed with the PC1 (10^8^ CFU or 10^11^ CFU per dose per mouse) with the antigens (days 0 and 14) and alone (days −1 and 13). The higher PC1 dose elicited a greater specific serum IgG2a response than IgG1 for both antigens and mice strains, indicating a Th1-biased humoral immune response. The Th1 bias was also observed at the cellular level with greater specific IFN-γ levels than IL-4 and IL-10 with both antigen types and mouse strains. With the particulate antigen, the lower dose of PC1 elicited a Th1 bias at the cellular level, but a balanced Th1/Th2 response at the systemic humoral level. With the soluble antigen, a strong Th1-biased response occurred at the cellular level while the systemic humoral response was Th2-biased. In conclusion, PC1 at the higher dose was an excellent Th1 adjuvant, which was unaffected by the nature of the antigen or the host’s genetic background.

## 1. Introduction

Many pathogens enter the body through the mucosal tissues. Oral vaccines can effectively combat such infections and can generate both mucosal and systemic immune responses [1]. However, generating robust immune responses via the oral route is particularly difficult as the vaccine has to withstand low pH, gastric enzymes and poor transport across the intestinal wall. Only a few oral vaccines are currently available for humans.

Oral adjuvants can protect against gastric degradation of the antigen, reduce the required dose of the antigen and abrogate oral tolerance whilst enhancing the desired immune responses. Adjuvants can also direct the Th1/Th2 nature of the immune response to a co-administered antigen. Mucosal adjuvants such as the heat labile toxin 4 and *Bacillus firmus* induce a Th1-biased immune response [2], whereas cholera toxin induces a more Th2-biased response [3]. Saponins generate a more balanced Th1/Th2 response and liposomes have been shown to also enhance Th1, Th2 and Th17 responses [4]. Host genetics, the nature of the antigen, the route of administration of the adjuvant-vaccine formulation and the dose of the antigen independently affect the nature and magnitude of immune responses [5,6,7,8].

*Lactobacillus* species can enhance mucosal and systemic immune responses to orally administered antigens [9,10]; however, *Lactobacillus* strains differ in their capacity to enhance responses to antigens [11,12,13]. Maassen et al. [14] showed that the Th1/Th2 nature of the immune response elicited by eight strains of *Lactobacillus* was strain-specific. Furthermore, *L. murines* and *L. casei* elicited Th2-biased humoral immunity whereas *L. reuteri* and *L. brevis* induced the expression of the Th1 cytokines when administered with TNP-CGG to mice [14]. *L. rhamnosus* HN001 induced mixed Th1/Th2 cytokine profiles in mice [15].

Previously, we showed that oral co-administration to mice of *L. fermentum* KLD or *L. fermentum* PC2 with a vaccine enhanced both systemic and mucosal immune responses [11] and conferred protection when subsequently challenged [16]. The strain used in the present study, PC1, was selected because of in vitro characteristics: survival in bile salts or low pH, superior adhesion to murine Peyer’s patches and enhanced cytokine IL-12 production by splenocytes (unpublished observations). The aims of this paper were to examine the adjuvanticity of PC1 and the impact of dosage levels, the nature of the antigen and the host genetics on the magnitude and nature of immune responses in a murine vaccine model.

## 2. Materials and Methods

### 2.1. Bacterial Cultures

*L. fermentum* PC1 (PC1) (FII511400) and *Salmonella enterica* Typhimurium (ST) (UNSW 078300) were obtained from the School of Biotechnology and Biomolecular Sciences (BABS), University of New South Wales (UNSW) culture collection. PC1 was grown in de Mann Rogosa Sharpe broth at 37 °C in an anaerobic chamber. The ST was grown aerobically at 37 °C in Tryptone Soya Broth with streptomycin (2 mg·mL^−1^). All experiments used 18 h secondary cultures of PC1 and ST inoculated (1%) into respective media.

### 2.2. Mice

Specific pathogen-free BALB/c and DBA/1 mice, six to eight weeks old, obtained from the Animal Research Centre (Perth, Australia) were used for breeding. Mice used in experiments were six- to eight-week-old BALB/c (*H-2^d^*) and DBA/ 1(*H-2^q^*) progeny mice bred and housed in the school animal facility in plastic cages (10 mice per cage) and fed ad libitum a commercial diet (Gordon’s Specialty Stockfeed, Sydney, Australia) and autoclaved water. All animal experiments were approved by the UNSW animal ethics committee.

### 2.3. Preparation of ST Cell Lysate (STCL)

A stationary-phase culture of *S. Typhimurium* was washed twice with phosphate buffered saline (PBS). The washed whole cell pellet was resuspended in PBS containing 1 mm phenylmethylsulfonylflouride (PMSF; Sigma, St. Louis, MO, USA) and calibrated to an absorbance of 0.8 at OD600 to give 1 × 10^9^ colony forming units (CFU) mL^−1^ of bacteria using a standard curve. The suspension was sonicated (Branson Ultrasonics, Danbury, CT, USA) for 10 mins at Duty Cycle 20% in an ice-bath. Following sonication, intact cells were removed by low-speed centrifugation (3000× *g*, 10 min). As this cell lysate was used for dosing mice, ELISA and the ex vivo assays an aliquot (100 μL) was plated onto Tryptone Soya agar (TSA) plates to check for sterility. STCL protein concentrations were determined using the Bradford protein assay as previously described [16]. Single use aliquots (0.5 mL) of STCL were stored at −80 °C until use.

### 2.4. In Vivo Experimental Protocol

Mice were randomised into groups of 10 and given one dose of PC1 orally on days −1, 0, 13 and 14. PC1 was used at two dosages; low (10^8^ CFU per dose per mouse) and high (10^11^ CFU per dose per mouse). On days 0 and 14, the vaccine antigen was orally administered. The particulate antigen was STCL (0.1 mg per dose per mouse) the soluble antigen was ovalbumin (OVA; 1 mg per dose per mouse; Sigma). All dosing were administered in PBS in a volume of 100 μL. Control mice, were given vaccine alone (vaccine control) or PBS (PBS control). On day 28, blood, intestinal fluid and spleens were collected as previously described [16] for immunoglobulin and cytokine analyses.

### 2.5. Antigen-Induced Lymphocyte Assay

Splenocytes (prepared as per [17]) were seeded at 2 × 10^5^ viable cells per well in 200 µL of RPMI complete medium. STCL or OVA was added to the well (final concentration 0.1 mg·mL^−1^) . Control wells contained medium alone or concanavalin A (ConA; 5 µg·mL^−1^; Sigma). Plates were incubated at 37 °C in the presence of 5% CO_2_ for three days. Supernatants were analysed directly for cytokine IL-4. Other cytokines were assayed after storage at −80 °C. Cytokines IL-4, IL-10 and IFN-γ were measured using ELISA with paired antibodies according to manufacturer’s instructions (Pharmingen, BD Biosciences, San Diego, CA, USA). Concentrations were calculated using standard curves and absorbance at 450 nm (Bench Mark microplate reader (Bio-Rad, Hercules, CA, USA).

### 2.6. Immunoglobulin Quantification

Antigen-specific IgG, IgG1, IgG2a and sIgA were measured in serum and intestinal fluid using ELISA in 96-well microtiter plates (Maxisorp Immunoplates, Nunc) as previously described [16]. The coating antigens (100 µL) were STCL (10 µg·mL^−1^) or OVA (5 µg·mL^−1^). Detection antibodies were peroxidase-conjugated affinity purified goat antibody to mouse IgG (γ) (1:1000) (Kiregaard and Perry Laboratories, USA), IgA (γ) (1:500) (Kiregaard and Perry Laboratories, USA), biotinylated rat anti-mouse IgG1 (κ) (1:1000) (Chemicon International) or biotinylated rat anti-mouse IgG2a (κ) (1:1000) (Chemicon International). For IgG1 and IgG2a, a Streptavidin-horseradish peroxidase conjugate (1:1000) (Silenus, Australia) step was included. Standard curves were generated using serum and intestinal fluid from mice hyper-immunised with STCL or OVA. These samples were arbitrarily assigned 1000 ELISA units (EU).

### 2.7. Statistical Analysis

Data were analysed by two-way ANOVA using XLSTAT statistical software (version 2009, Addinsoft, New York, NY, USA). Differences between means of groups were determined using the REGWQ test. *p*-Values < 0.05 were considered significant.

## 3. Results

### 3.1. STCL-Specific sIgA in Intestinal Fluid

The particulate antigen, STCL, used at a sub-optimal dose allowed for the impact of the experimental parameters on the immune response to be demonstrable. Mucosal antibody responses were measured by examining antigen-specific sIgA levels in intestinal fluid. The inclusion of PC1 in the vaccine schedule resulted in significantly higher sIgA levels (*p* < 0.05, compared to vaccine controls; Table 1). Slightly higher sIgA levels were detected in the BALB/C mice compared to those detected in the DBA/1 mice. The high dose of PC1 produced slightly greater levels of sIgA than the low dose.

### 3.2. STCL-Specific Antibodies in Sera

**Figure 1:**
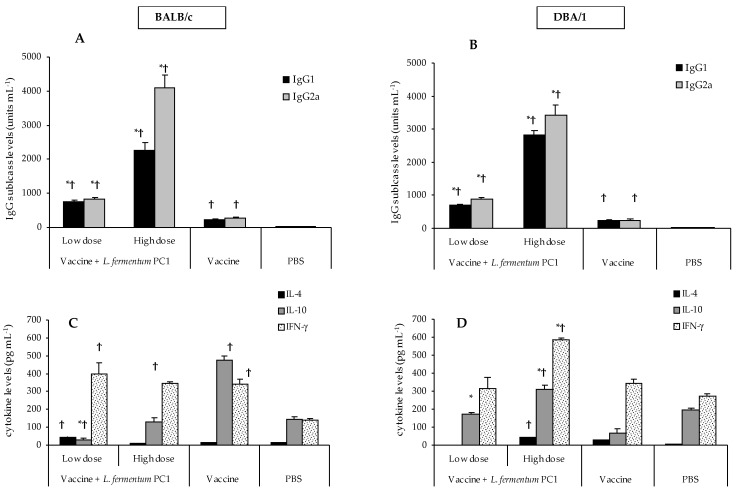
STCL-specific immune responses in BALB/c and DBA/1 mice. The mice were given STCL vaccine (0.1 mg per dose per mouse) with either a low dose of PC1 (10^8^ CFU per dose per mouse) or a high dose (10^11^ CFU per dose per mouse) on days 0 and 14. In addition, the mice received PC1 alone on days −1 and 13. The vaccine group received the STCL vaccine alone. PBS group received PBS as the negative control. On day 28, blood samples were collected, and assayed for IgG subclasses in BALB/c (**A**) and DBA/1 (**B**) mice. Cytokine levels in spleens were quantified in the BALB/c (**C**) and DBA/1 (**D**) mice. Antibody values are expressed as units mL^−1^ + SEM. Cytokine results are expressed as the mean + SEM. There were 10 mice in each group. Results from one representative experiment out of two are presented. * *p* < 0.05 compared to vaccine group; ^†^ *p* < 0.05 compared to PBS group.

The vaccine alone resulted in low levels of both IgG1 and IgG2a in both mouse models (Figure 1A,B), indicative of a low but balanced Th1/Th2 serum humoral immune response. BALB/c mice elicited high levels of both IL-10 and IFN-γ, with little IL-4 production (Figure 1C), indicative of a balanced Th1/Th2 response. However, in DBA/1 mice high levels of IFN-γ with little IL-4 and IL-10 (Figure 1D) were demonstrated, indicating a clear Th1-biased immune response.

The high dose of PC1 elicited significantly higher levels of IgG subclasses (*p* < 0.05, compared to vaccine and PBS controls) in both BALB/c and DBA/1 mice; IgG2a was significantly greater than IgG1 (*p* < 0.05), signifying a Th1-biased serum humoral immune response (Figure 1A,B). The cytokine results mirrored the IgG bias seen in serum, with higher IFN-γ than IL-4 and IL-10 levels in both mouse models (Figure 1C,D).

The low dose of PC1 also resulted in significant augmentation of specific IgG subclass levels compared to the vaccine groups for both mouse models; however, the IgG1 and IgG2a subclass levels were comparable, indicating a balanced Th1/Th2 serum humoral response (Figure 1A,B). The collective IgG1 and IgG2a responses were approximately four-fold less than those observed for the high dose of PC1 in both mice strains. Interestingly, the cytokine profiles demonstrated little (BALB/c mice) or no IL-4 (DBA/1) and higher levels of IFN-γ, indicative of a Th1-biased cellular response in both mouse models (Figure 1C,D).

### 3.3. OVA-Specific sIgA in Intestinal Fluid

In the BALB/c and the DBA/1 mice strains vaccinated with OVA, both the low and the high doses of PC1 significantly enhanced mucosal sIgA levels (*p* < 0.05) compared to vaccine controls (Table 2). The high dose of PC1 resulted in slightly higher levels of sIgA compared to the low dose for both mouse groups. With the PC1 dosage, statistically greater (*p* < 0.05) levels of antigen-specific sIgA were noted in the BALB/c mice compared to the DBA/1 mice (Table 2). Interestingly, the OVA antigen yielded lower sIgA levels in the vaccine and PC1 groups compared to their STCL counterparts (Table 1 and Table 2).

### 3.4. OVA-Specific Antibodies in Sera

**Figure 2:**
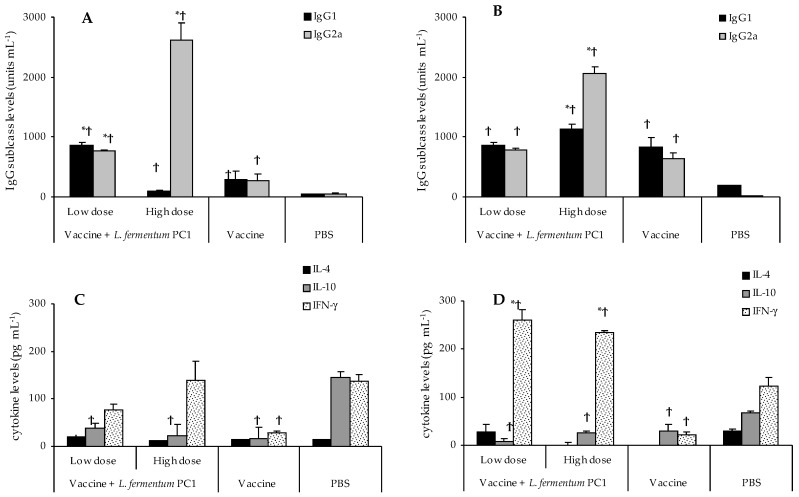
OVA-specific immune responses in BALB/c and DBA/1 mice. The mice were given OVA (1 mg per dose per mouse) with a PC1 low dose (10^8^ CFU per dose per mouse) or high dose (10^11^ CFU per dose per mouse) on days 0 and 14. In addition, the mice received PC1 alone on days −1 and 13. The vaccine group received the OVA vaccine alone. PBS group received PBS as the negative control. On day 28, serum samples were collected and assayed for IgG subclasses in BALB/c (**A**) and DBA/1 (**B**) mice. Cytokines in spleens were also quantified in the BALB/c (**C**) and DBA/1 (**D**) mice. Antibody values are expressed as units mL^−1^ + SEM. Cytokine results are expressed as the mean + SEM. There were 10 mice in each group. Results from one representative experiment out of two are presented. * *p* < 0.05 compared to vaccine group; ^†^ *p* < 0.05 compared to PBS group.

Significantly less serum IgG1 and IgG2a levels were obtained in vaccine-only mice than in those mice given PC1. The levels of IgG1 and IgG2a were comparable and indicative of a balanced Th1/Th2 response (Figure 2A,B).

The high dose of PC1 with the soluble antigen OVA produced significantly higher levels of OVA-specific IgG2a than the vaccine alone in both mouse groups, with significantly greater IgG2a than IgG1 levels, indicative of Th1-biased systemic humoral immunity (Figure 2A,B). This Th1/Th2 response was mirrored in the cell-mediated immunity where the splenic cytokine profiles in both mice exhibited greater levels of IFN-γ compared to IL-4 and IL-10 (Figure 2C). Both mice produced significantly greater IgG2a than IgG1 (Figure 2A,B), indicative of a Th1 bias on the systemic humoral immunity. The cytokine profiles also showed a Th1 bias in both mice with significantly more IFN-γ (*p* < 0.05, compared with vaccine group) and a suppression of IL-4 and IL-10 (Figure 2C,D).

The low dose of PC1 with the soluble antigen OVA induced significantly more of the IgG subclasses in both mice compared to the vaccine group (*p* < 0.05; Figure 2A,B). The IgG1 and IgG2a levels in these mice were comparable, indicative of a balanced Th1/Th2 humoral response (Figure 2A,B). However, the splenic cytokine profiles in BALB/c mice showed slightly higher levels of IFN-γ compared to IL-4 and IL-10 (Figure 2C). In DBA/1 mice, the Th1 bias was more pronounced with significantly higher levels of IFN-γ than IL-4 and IL-10 (Figure 2D).

### 3.5. Differential Immune Responses to STCL and OVA

A comparison of the IgG subclass levels for the two antigens revealed that both mice given the high dose of PC1 with the particulate STCL had significantly higher levels of specific IgG1 and IgG2a than was obtained for those given the soluble OVA antigen (Figure 1A,B compared to Figure 2A,B). In addition, the inclusion of PC1 generated higher IFN-γ levels for both antigens (Figure 1C,D and Figure 2C,D) in both mice strains, indicating a Th1-biased cell mediated immunity.

## 4. Discussion

A murine vaccine model was used to study the adjuvanticity of *L. fermentum* PC1. The magnitude of the resultant immune response was dependent on the type of antigen, the adjuvant dose and the host genetics. The Th1/Th2 nature of the response was dependent primarily on the dose of the PC1. The high dose of PC1 elicited robust Th1-biased antigen-specific humoral and cell-mediated immune responses, independent of the antigen type and host genetics. Furthermore, the particulate STCL antigen elicited greater total IgG subclass production than was obtained for the soluble OVA antigen.

The lack of immunogenicity of mucosally administered soluble antigens has been a major obstacle in the development of many oral vaccines [18]. Adjuvants CpG-ODN and cholera toxin in oral vaccine programs have been shown to abrogate oral tolerance and elicit robust OVA-specific Th2-biased humoral responses [19,20]. In the present study, both STCL and OVA antigens, when given with a high dose of PC1, elicited significantly greater Th1-specific humoral and cellular immune responses in both BALB/c and DBA/1 mice compared to the vaccine controls. In a previous study [13], the Th1/Th2 bias was skewed towards a definite Th1-biased immune response in Th2-prone BALB/c mice when a *Lactobacillus brevis* strain was co-administered with OVA. This *L. brevis* strain demonstrated a stronger Th1-biasing ability than the other strains tested, confirming that this capacity is strain-specific for lactobacilli. This finding is also in agreement with a study by Maassen et al. [14] who reported that two of eight probiotic strains resulted in the elevation of Th1 cytokines in the gut when co-administered with a soluble antigen in BALB/c mice.

BALB/c, widely used as a Th2-biased model, and DBA/1 mice, which exhibit more Th1-biased responses, were used to examine the influence of host genetics on the immune responses. These mouse strains express different MHC-1 molecules and process antigens differently [21]. Both BALB/c and DBA/1 mice produced significantly lower levels of OVA-specific gastrointestinal sIgA than the mice given OVA with PC1, and both elicited very weak systemic responses to OVA and STCL antigens with Th1/Th2 balanced serum IgG levels in the absence of the PC1. This Th1/Th2 balance was also observed in the cell-mediated immune responses in the OVA vaccine groups; however, both mouse strains given the STCL vaccine had higher levels of IFN-γ, indicating a more Th1-biased cell-mediated response. Both mice strains given the high dose of PC1 exhibited robust Th1-biased serum IgG and cell-mediated immune responses for both antigens. It was noted that the mice given the low dose of PC1 did not skew the Th1/Th2 balance of the vaccine-induced serum IgG responses for either antigen, but did mount a more Th1-biased cell-mediated immune response for both antigens. This finding is in agreement with another study that found that heat-killed *Brucella abortus*, a potent Th1 inducer, also generated a Th1-predominant immunity in Th2-biased BALB/c mice [22]. In the present study, for each antigen, both doses of PC1 elicited the same Th bias and the magnitude of the responses was comparable for both mouse strains. However, it was noted that although both mouse strains demonstrated the same Th bias, the levels of individual cytokines and IgG subclasses differed. Others have demonstrated differential systemic immune responses to orally administered *Salmonella* flagellin antigen in other mouse strains [23]. A study using *Streptococcus mutans* surface protein reported that BALB/c mice elicited a more robust immune response compared to DBA/1 mice [21]. However, the present study differs in the mouse strains and antigen-adjuvant formulations used and therefore does not allow for comparisons to be made.

Oral administration of PC1 enhanced the production of gastrointestinal mucosal sIgA responses and significantly enhanced humoral and cellular-mediated immune responses to both OVA and STCL antigens. Although both mouse groups had comparable serum and cell-mediated responses, gastrointestinal responses differed in antigen-specific immunoglobulins. This was possibly due to a higher abundance and diversity of IgAs which have been shown to correlate with the production of specific sIgA in BALB/c mice [24].

In the present study, the high dose of PC1 resulted in greater augmentation of antigen-specific systemic and mucosal humoral responses in both mouse strains, compared to the low dose. Moreover, PC1 at the high dose elicited a robust Th1 humoral and cellular response characterised by high levels of IgG2a (compared to IgG1) and IFN-γ (relative to IL-4 and IL-10), regardless of the nature of the antigen and genetic background of the host. However, PC1 adjuvanticity at the lower dose was affected by the nature of the co-administered antigen but not by the host genetics. The particular co-administered antigen affected the immune-enhancing effects of the low dose of PC1. When used with the particulate antigen STCL, the systemic humoral response was slightly Th1-biased (more IgG2a than IgG1, but not significant), and with OVA antigen, the systemic humoral response was slightly skewed to Th2 (more IgG1 than IgG2a, but not significantly different). Surprisingly, at the cellular level, more Th1-biased responses, evidenced by greater production of IFN-γ than IL-4 and IL-10, were noted for both antigens and in both mouse models. It is difficult to compare our results with others as different groups have reported very different immune-enhancing properties for the same probiotic strains using different dosing regimens, antigens and hosts. However, two studies have used a similar vaccine program with two *Lactobacillus* species. Different doses of *Lactobacillus rhamnosus* GG with human rotavirus vaccine (HRV) yielded differential effects on specific cell-mediated immunity in pigs. A high dose of *L. rhamnosus* GG induced significantly increased HRV-specific IFN-γ T cell responses, whilst a low dose failed to elevate IFN-γ levels [25]. When *L. acidophilus* NCFM was used in the same vaccine program, the lower dose significantly increased HRV-specific IFN-γ T cell responses whilst the higher dose increased the frequency of T regulatory cells [26]. It appears that the effect of the adjuvant dosage on the specific immunity is dependent on the *Lactobacillus* strain and the antigen used.

The adjuvanticity of probiotics is strain-dependent. Previously, we reported that *L. fermentum* PC2 significantly enhanced STCL-specific humoral responses whilst *L. acidophilus* L10, which exhibited excellent immune-modulatory properties in vitro, did not elicit very robust specific responses in vivo [16]. Strains with similar in vitro probiotic properties have been shown to induce very different mucosal IgA levels in vivo [27]. Link-Amster et al. [9] reported augmented vaccine-specific serum IgA levels in subjects given *L. acidophilus* La1 and *Bifidobacterium* Bb12 with *Salmonella typhi* Ty21a oral vaccine. Likewise, a human study found that the consumption of a commercially available fermented drink, Actimel^®^ (Danone, Paris, France), which contains, amongst other probiotics, the strain *Lactobacillus casei DN*-114 001, during an influenza vaccination program led to the significant increase in the level of specific influenza antibodies [28]. Another influenza vaccine study documented that the two strains used, *Bifidobacterium lactis* BB-12 and *Lactobacillus casei* 431, both augmented specific antibody responses [29]. Others showed that an oral cholera vaccination varied in the kinetics and magnitude of the specific mucosal and serum humoral response for each of seven probiotics strains that were tested [30]. Probiotic strains, *Bifidobacterium lactis* Bl-04 and *Lactobacillus acidophilus* La-14, increased early specific serum immunoglobulin levels whilst *L. acidophilus* NCFM exhibited increased late specific serum IgA and IgM levels.

At this stage, it is unknown what component(s) of PC1 contribute to the immune enhancement seen in this study. However, a number of components, including the cell wall, exopolysaccharides, lipoteichoic acid and other components of other probiotic strains, have been reported to activate immune cells such as macrophages and dendritic cells, resulting in differential Th1 and Th2 immune responses (reviewed in [31]).

The fact that PC1 directed Th1 differentiation of antigen-specific immune responses has significant implications. Newborns and young infants are at elevated risk of infections that require the induction of strong Th1 immune responses because of the intrinsic Th2 bias of human neonates [32,33]. The efficacy of conventional vaccines is compromised in newborns; however, the inclusion of PC1 with existing vaccines could evoke protective Th1 responses without having to redesign the vaccines.

## 5. Conclusions

In this study, PC1 was shown to function efficiently as an oral adjuvant in a murine model. The magnitude and nature of the specific immune response was influenced by the dose of adjuvant, type of co-administered antigen and host genetics. A high dose of PC1 elicited a robust Th1 response independent of nature of antigen and genetic background of the host.

## Figures and Tables

**Table 1:** *S*. Typhimurium cell lysate (STCL)-specific secretory immunoglobulin A (sIgA) in intestinal fluid after vaccination of two different strains of mice, namely BALB/c and DBA/1. The mice were given STCL vaccine (0.1 mg per dose per mouse) with either a low dose of *L. fermentum* PC1 (10^8^ CFU per dose per mouse) or a high dose (10^11^ CFU per dose per mouse) on days 0 and 14. In addition, the mice received PC1 alone on days −1 and 13. The vaccine group received the STCL vaccine alone. The phosphate buffered saline (PBS) group received only PBS as the negative control. On day 28, intestinal fluid was collected and assayed for sIgA.

GROUPS	BALB/c	DBA/1
*L. fermentum* PC1—Low dose	3320.26 ± 458.48 *^,†^	2563.58 ± 563.58 *^,†^
*L. fermentum* PC1—High dose	3558.48 ± 598.49 *^,†^	2687.71 ± 472.94 *^,†^
Vaccine alone	847.15 ± 158.97 ^†^	1245.84 ± 256.59 ^†^
PBS control	156.35 ± 35.56	163.95 ± 49.47

Results from one representative experiment out of two are presented and expressed as colony forming units (CFU). Antibody values are expressed as units mL^−1^ ± SEM. There were 10 mice in each group. * *p* < 0.05 compared to vaccine group; ^†^ *p* < 0.05 compared to PBS group.

**Table 2:** Effect of PC1 dosage on levels of OVA-specific sIgA in intestinal fluid. The mice were given OVA (1 mg per dose per mouse) with a PC1 low dose (10^8^ CFU per dose per mouse) or high dose (10^11^ CFU per dose per mouse) on days 0 and 14. In addition, the mice received PC1 alone on days −1 and 13. The vaccine group received the OVA alone. PBS control mice received PBS as the negative control. On day 28, intestinal fluids were collected and assayed for sIgA.

GROUPS	BALB/c	DBA/1
*L. fermentum* PC1—Low dose	2587.48 ± 742.63 *^,^^†^	1985.47 ± 596.81 *^,^^†^
*L. fermentum* PC1—High dose	2758.41 ± 563.49 *^,^^†^	1987.56 ± 695.32 *^,^^†^
Vaccine alone	685.71 ± 217.32 ^†^	658.14 ± 147.29 ^†^
PBS control	98.47 ± 45.69	49.36 ± 34.83

Results from one representative experiment out of two are presented. Antibody values are expressed as units mL^−1^ ± SEM. There were 10 mice in each group. * *p* < 0.05 compared to vaccine group; ^†^ *p* < 0.05 compared to PBS group.
